# Dolutegravir Interactions with HIV-1 Integrase-DNA: Structural Rationale for Drug Resistance and Dissociation Kinetics

**DOI:** 10.1371/journal.pone.0077448

**Published:** 2013-10-16

**Authors:** Felix DeAnda, Kendra E. Hightower, Robert T. Nolte, Kazunari Hattori, Tomokazu Yoshinaga, Takashi Kawasuji, Mark R. Underwood

**Affiliations:** 1 Chemical Sciences, GlaxoSmithKline, Research Triangle Park, North Carolina, United States of America; 2 Biological Sciences, GlaxoSmithKline, Research Triangle Park, North Carolina, United States of America; 3 Discovery Chemistry, Shionogi & Co., Ltd., Osaka, Japan; 4 Infectious Diseases, Shionogi & Co., Ltd., Osaka, Japan; 5 Chemistry Infectious Diseases, Shionogi & Co., Ltd., Osaka, Japan; 6 Medicines Development Infectious Diseases, GlaxoSmithKline, Research Triangle Park, North Carolina, United States of America; McGill University AIDS Centre, Canada

## Abstract

Signature HIV-1 integrase mutations associated with clinical raltegravir resistance involve 1 of 3 primary genetic pathways, Y143C/R, Q148H/K/R and N155H, the latter 2 of which confer cross-resistance to elvitegravir. In accord with clinical findings, in vitro drug resistance profiling studies with wild-type and site-directed integrase mutant viruses have shown significant fold increases in raltegravir and elvitegravir resistance for the specified viral mutants relative to wild-type HIV-1. Dolutegravir, in contrast, has demonstrated clinical efficacy in subjects failing raltegravir therapy due to integrase mutations at Y143, Q148 or N155, which is consistent with its distinct in vitro resistance profile as dolutegravir’s antiviral activity against these viral mutants is equivalent to its activity against wild-type HIV-1. Kinetic studies of inhibitor dissociation from wild-type and mutant integrase-viral DNA complexes have shown that dolutegravir also has a distinct off-rate profile with dissociative half-lives substantially longer than those of raltegravir and elvitegravir, suggesting that dolutegravir’s prolonged binding may be an important contributing factor to its distinct resistance profile. To provide a structural rationale for these observations, we constructed several molecular models of wild-type and clinically relevant mutant HIV-1 integrase enzymes in complex with viral DNA and dolutegravir, raltegravir or elvitegravir. Here, we discuss our structural models and the posited effects that the integrase mutations and the structural and electronic properties of the integrase inhibitors may have on the catalytic pocket and inhibitor binding and, consequently, on antiviral potency in vitro and in the clinic.

## Introduction

**Figure 1:**
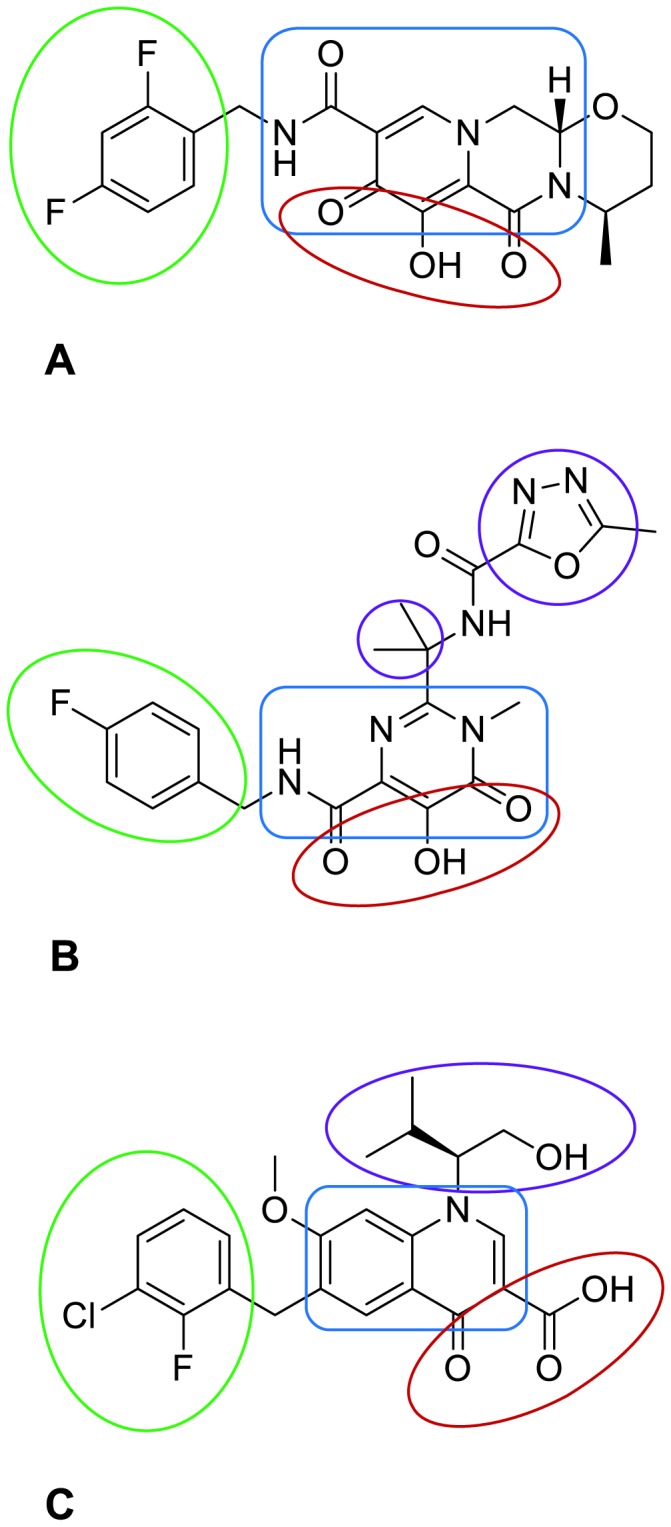
2D structures of (A) dolutegravir, (B) raltegravir and (C) elvitegravir. Red ovals encircle the oxygen atoms that chelate the divalent metal cations in the active site; green ovals encircle the halobenzyl groups; and blue boxes encircle the approximate regions of the scaffolds that can accommodate positive charge after chelation of the metals. The purple circles at (B) encircle raltegravir’s gem-dimethyl (small circle) and oxadiazole groups, and the purple oval at (C) encircles elvitegravir’s 1-hydroxymethyl-2-methylpropyl group.

HIV-1 integrase (IN) is required for viral cDNA integration into the host cell genome, an essential step in the HIV life cycle. First, IN catalyzes the cleavage of a GT dinucleotide from the 3′ end of each viral long terminal repeat (LTR) that is downstream from a conserved CA dinucleotide (3′ processing). Next, the enzyme catalyzes the concerted insertion of the 2 processed 3′ ends into opposite strands of the host target DNA 5 base pairs apart from each other by a direct trans-esterification reaction (strand transfer). Because of the vital role that IN plays in HIV replication, the enzyme is an attractive therapeutic target. Extensive research efforts have led to the discovery and development of the IN inhibitors, raltegravir (RAL) and elvitegravir (EVG), and the new IN inhibitor, dolutegravir (DTG) (Figure 1), all of which have demonstrated efficacy in clinical studies by preferentially inhibiting the strand transfer activity of IN [1-3].

Clinical RAL resistance is associated with 3 primary genetic pathways that involve IN mutations at Y143, Q148 or N155, whereas EVG resistance is associated with mutations at Q148 or N155 as well as T66, E92, T97 or S147 [4-7]. In subjects who have failed RAL therapy with RAL-resistant HIV-1, DTG has demonstrated greatest efficacy in those harboring HIV-1 with Y143 or N155 pathway mutations, and more limited responses when Q148 pathway viruses with additional secondary mutations are present [8]. In accord with in vivo results, in vitro drug resistance profiling studies with wild-type and site-directed IN mutant viruses have shown that DTG has a distinct profile compared with those of RAL and EVG [7]. Indeed, DTG’s antiviral activity against the single IN mutants mentioned remains comparable to its activity against wild-type HIV-1 and has only a 2.6-fold increase in resistance against the Q148H/G140S IN mutant virus compared with >130- and >890-fold increases for RAL and EVG, respectively [7]. Dolutegravir’s wild-type activity against the single IN mutants is consistent with a higher barrier to resistance and suggests that multiple mutations are needed to confer resistance [7,8]. Consistent with this concept, there was no evidence of treatment-emergent resistance in patients with virologic failure on DTG in the ART-naive, IN strand transfer inhibitor (INSTI)-naive SPRING-2 study [3]. In addition, in the ART-experienced, INSTI-naive SAILING study [9], 16 patients in the RAL group had typical treatment-emergent RAL resistance with high fold-changes to RAL, and 2 patients receiving DTG developed the IN substitution R263K, which conferred fold-changes of <2. Kinetic studies [10] involving IN inhibitor dissociation from wild-type and mutant IN-viral DNA complexes show that DTG also has a distinct off-rate profile compared with those of RAL and EVG. The dissociative half-lives (t_1/2_) of DTG were found to exceed those of RAL and EVG by 8- to 38-fold, suggesting that the prolonged binding of DTG to the nucleoprotein complexes may be a significant contributing factor to its distinct resistance profile.

To gain structural insights into the drug resistance and dissociation kinetics profiles of DTG, RAL and EVG, molecular models of wild-type and mutant HIV-1 IN in complex with viral DNA and IN inhibitor are needed. Although significant time and resources have been dedicated to the effort, there are currently no crystal structures of full-length HIV-1 IN available, or of it bound to viral DNA and an inhibitor. There are, however, published structures of the individual IN domains and of the catalytic core in combination with the N- or C-terminal domain, which have been used to build numerous IN-DNA models [11-15] supplemented with structural data from related retroviral IN enzymes (eg, avian sarcoma virus IN) or bacterial transposases (eg, Tn5 transposase) as well as protein-DNA cross-linking and protein footprinting data [16-21]. Owing to the work of Hare et al [22-24], crystal structures of the wild-type, S217H and N224H prototype foamy virus (PFV) intasomes are now available, and many of these are in complex with 1 of several IN inhibitors, including DTG, RAL and EVG, providing the first molecular views into the likely architecture of the HIV-1 IN catalytic site when bound to viral DNA and inhibitor. Using the PFV intasome as a template, HIV-1 intasome models have been constructed to gain insights into the mechanism of IN inhibition, RAL and EVG resistance [25,26], and cross-resistance to MK-2048 [6]. In our efforts to structurally rationalize DTG’s distinct resistance and dissociation kinetics profiles, we constructed wild-type, Q148R, Q148K, Q148H/G140S and N155H HIV-1 IN models in complex with U5 LTR DNA using carefully selected structural data from wild-type and mutant PFV intasomes as well as Tn5 transposase to model key missing active-site elements from a selected structure of the HIV-1 IN catalytic core. Dolutegravir, RAL or EVG were then docked into the active sites of most of these IN-DNA models. In this report, we discuss the insights provided by our molecular models.

## Materials and Methods

Wild-type, Q148R, Q148K, Q148H/G140S and N155H models of the HIV-1 IN dimeric catalytic core were constructed starting from the HIV-1 IN structure in 2B4J [27], which was taken from the RCSB Protein Data Bank (PDB; www.pdb.org) [28]. While various catalytic core structures were considered, none offered a clear advantage. 2B4J was selected because of the 2 molecules of LEDGF/p75 that we speculated might aid in “docking” the viral DNA substrate. The models’ active-site loop residues, G140-G149, portions of the α4 helix and the Mg^2+^-bound conformations of D64, D116 and E152 for chain A were modeled based on selected PFV IN structures. For the wild-type HIV-1 IN model, the active-site loop was modeled from that of PFV IN in PDB entry 3OYA [23], specifically residues S209-G218. In modeling the metal-bound conformation of E152, the PFV IN template was extended by approximately the first helical turn of the α4 helix to include residues K219-E221, which correspond to HIV-1 IN residues V150-E152. The metal-bound conformations of HIV-1 IN residues D64 and D116 were also modeled based on their PFV IN counterparts, residues D128 and D185, respectively. For the Q148R HIV-1 IN model, residue Q148 of the wild-type IN model was mutated to Arg, then its rotameric state set to match that of the equivalent Tn5 transposase residue, R322, from PDB entry 1MUS [29]. This positioned Q148R to form an ionic interaction with the side chain of E152 similar to the equivalent Tn5 transposase residues R322 and E326, respectively. The Q148K HIV-1 IN model was built in similar fashion to the Q148R IN model. The Q148H/G140S HIV-1 IN model was constructed following the same approach used to build the wild-type IN model. Here, however, the active-site loop, first helical turn of the α4 helix and Mg^2+^-bound conformations of D64, D116 and E152 were modeled from those of S217H PFV IN in PDB entry 3S3N [24]. The use of S217H PFV IN was important in modeling the structural disturbances expected at the active-site loop from the Q148H/G140S substitutions. The N155H HIV-1 IN model was constructed with its active-site loop and a significant portion of the α4 helix modeled from those of N224H PFV IN in PDB entry 3S3O [24]. More specifically, the PFV IN template included residues S209-K228 to model HIV-1 IN residues G140-K160, thus capturing the metal-bound conformation of HIV-1 IN residue E152 as well as the structural disturbances expected at the α4 helix due to the N155H substitution. The metal-bound conformations of residues D64 and D116 were also modeled based on those of the selected PFV IN mutant.

The wild-type HIV-1 IN model was used to construct 3 IN-DNA-inhibitor complexes. For each complex, an appropriate PFV DNA template was chosen for HIV-1 DNA based on the inhibitor to be docked at the catalytic site as the inhibitor is predicted to impact the conformation of the terminal 3′ adenylate. The IN-DNA model in complex with RAL was assembled first starting with the addition of a 3′ processed, HIV-1 U5 LTR end modeled from the PFV U5 LTR end in 3OYA. Additional analysis of the electron density generated from the deposited 3OYA structure factors suggested, however, that an alternate conformation of the 3′ adenylate may exist; thus, 2 conformations were modeled for this nucleotide in HIV-1 DNA. Given that PFV DNA was docked onto the IN model by the superimposition of the HIV-1 and PFV IN catalytic cores, the PFV U5 nucleotides were simply substituted with those of HIV-1 (Figure 1A, Supplementary Experimental Procedures in File S1). The rotameric states of T66, S153 and K156 were reassessed in the presence of HIV-1 DNA and then modified to form energetically favorable interactions with the DNA. Two Mg^2+^ ions were positioned at the IN catalytic site guided by those of 3OYA. Raltegravir was then manually docked into the active site with its binding mode set nearly identical to that seen in 3OYA. To complete the octahedral distribution of coordinating ligands about each metal cation, 3 water molecules were added with their placement guided by those that coordinate the Mg^2+^ ions in 3OYA.

The HIV-1 IN-DNA models in complex with EVG and DTG were constructed in similar fashion to the IN-DNA-RAL model but without in-house modifications to the molecular templates as with 3OYA above. The IN-DNA-EVG model was assembled based on the EVG-bound, PFV intasome in PDB entry 3L2U [22], while the IN-DNA-DTG model was assembled based on the DTG-bound, PFV intasome in PDB entry 3S3M [24]. A Q148R and Q148K IN-DNA model were built using the PFV DNA from 3S3M, however an inhibitor was purposely not docked into their active sites.

HIV-1 IN-DNA complexes were also constructed using the Q148H/G140S and N155H IN models, but here only DTG was docked into the active site. The Q148H/G140S IN-DNA-DTG model was assembled based on the DTG-bound, PFV S217H intasome in 3S3N, with the exception of the two 3′ adenylate conformations published for the PFV DNA. The conformer with the adenine near PFV nucleotide C16 was drawn into question given that its N7 atom and the O3 atom of PFV nucleotide C15 were ~2.8 Å apart, and thus at a distance that is not chemically sensible for H-bond acceptors. For the alternate conformer, our analysis suggested that the conformations of the 3′ adenylate modeled in 3S3M and 3S3O were more consistent with the electron density observed in 3S3N. Given these observations, we elected to use a 3′ adenylate conformation consistent with those from 3S3M and 3S3O for our model since the nucleotide was clearly defined in the latter structures and is also expected to make similar favorable interactions with DTG. Lastly, the N155H IN-DNA-DTG model was assembled based on the DTG-bound, PFV N224H intasome in 3S3O.

Comprehensive details on the construction and structural refinement of the different HIV-1 IN models and IN-DNA complexes with DTG, RAL or EVG bound at the catalytic site are provided in Supplementary Experimental Procedures in File S1, together with the amino acid sequence and structural alignments of HIV-1 and PFV IN (Figure S1), and the analysis of the PFV-related structure factors.

## Results

Intuitively, PFV IN is a logical template for an HIV-1 IN model given that these retroviral enzymes are structurally and mechanistically related. Krishnan et al [25] were the first to construct an HIV-1 intasome model from the 3L2S PFV intasome structure; Goethals et al [6] and Johnson et al [30] followed by extending the modeling of the HIV-1 intasome to include mutant enzymes. Unlike the nearly full-length HIV-1 IN models built by these investigators, we instead constructed models of the wild-type and mutant IN dimeric catalytic core as the spatial arrangement of the N- and C-terminal domains surrounding the catalytic core may be different between HIV-1 and PFV IN. Support for this view lies in the following. Firstly, the sequence identity between the enzymes’ common domains is only ~19% (Figure S1A). Secondly, PFV IN has 2 extensive amino acid insertions compared with HIV-1 IN—a 50-residue segment preceding the N-terminal domain and a 29-residue segment between the catalytic core and C-terminal domains. Thirdly, the PFV IN linkers between the N-terminal, catalytic core and C-terminal domains have mostly extended conformations signifying a high degree of enzyme flexibility. Nevertheless, the superimposition of the isolated HIV-1 and PFV IN catalytic cores shows that these domains share a very similar RNase H-like fold suggesting that this PFV domain may be a reasonable template for that of HIV-1 IN.

The approach we undertook in constructing our wild-type IN model involved using segments from a select PFV IN template to model those missing from the HIV-1 IN structure in 2B4J. Our Q148H/G140S and N155H IN models were built in a similar fashion using segments from the S217H and N224H PFV IN crystal structures, respectively, to capture the structural disturbances induced by the mutant residues. This is in contrast to Goethals et al, who built their mutant IN models by manually altering residues, and Johnson et al, who modeled only the HIV-1 IN mutant residues from their selected PFV templates. In constructing our Q148R IN model, we mutated residue Q148 of the HIV-1 IN model to Arg and then modified its rotameric state to match that of the corresponding residue from a selected Tn5 transposase template. For our wild-type and mutant HIV-1 IN-DNA models, a unique aspect of our work involved using the PFV DNA molecules associated with the selected IN templates to build distinct HIV-1 DNA models to capture the impact that the bound IN inhibitor may have on the conformation of the terminal 3′ adenylate.

**Figure 2:**
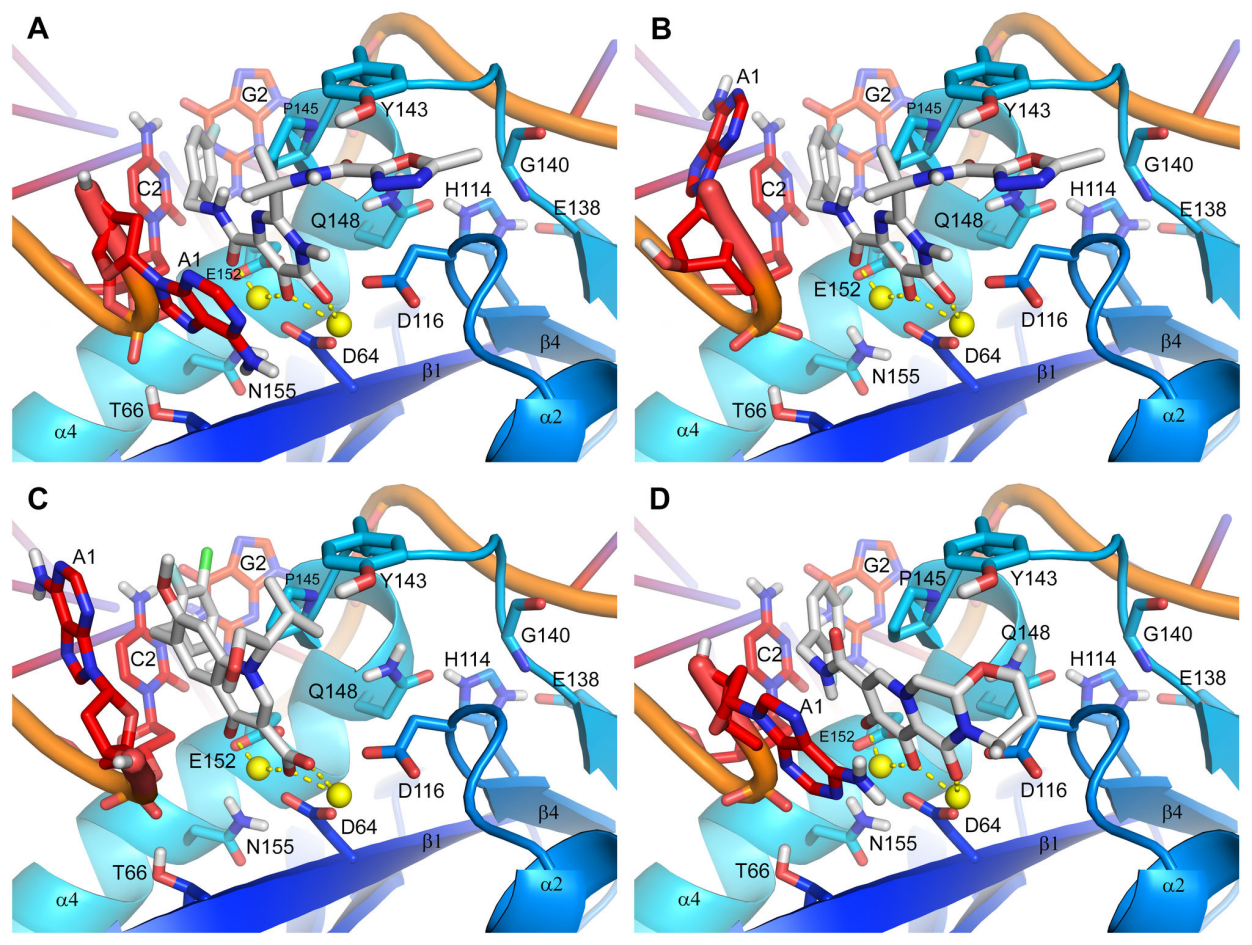
Structural models of HIV-1 integrase with U5 LTR DNA and (A, B) raltegravir, (C) elvitegravir or (D) dolutegravir. For raltegravir, the terminal 3′ adenylate is depicted in 2 distinct conformations: panel 2A shows the published conformer and panel 2B shows an alternative conformer that is also consistent with the observed electron density. Raltegravir, elvitegravir and dolutegravir are in stick representation with carbon, nitrogen, oxygen, fluorine and chlorine atoms colored gray, blue, red, cyan and green, respectively. A select subset of amino acids and nucleotides is depicted and labeled with residue type and number (numbering schemes as listed in Figure S1); all residues are in stick representation with carbon atoms colored by secondary structure/chain and nitrogen and oxygen atoms colored blue and red, respectively. The Mg^2+^ ions are represented as small yellow spheres with coordinate bonds to the inhibitors depicted as dashed yellow lines.

The catalytic sites of the wild-type HIV-1 IN-DNA models in complex with RAL, EVG and DTG are illustrated in Figures 2A to 2D. Consistent with previous reports [31,32] is that the 3 IN inhibitors share common pharmacophoric features essential for binding to the active site. One feature is the trio of oxygen atoms (Figure 1) that coordinate the Mg^2+^ ions at the base of the catalytic pocket. The 5-hydroxyl group of RAL and the 7-hydroxyl group of DTG are modeled negatively ionized, which is necessary for the formation of coordinate bonds to the metal cations; the carboxylic acid of EVG is, of course, negatively ionized. A second common feature is their halobenzyl groups (Figure 1), which π-stack with nucleotide C2 (numbering scheme as listed in Figure S1B) of the transferred DNA strand near the 3′ processed end; these groups are also in van der Waal contact with residue P145, the alkyl portion of E152’s side chain and nucleotide G2 of the non-transferred DNA strand. A distinctive pharmacophore of RAL, however, is its 1,3,4-oxadiazole, which π-stacks with residue Y143 (Figures 2A-B). In contrast, EVG and DTG make only limited van der Waals contact with Y143, EVG mostly through its isopropyl group laying in proximity to the Cα, Cβ and C2 atoms of Y143, (Figure 2C) and DTG mostly through the oxygen atom and adjacent methylene of its tetrahydro-oxazine group laying in proximity to the C2, C3 and C4 atoms of Y143 (Figure 2D). Note that a unique attribute of DTG is the “streamlined” architecture of its metal-chelating scaffold as DTG spans the width of the binding pocket from the β4-α2 connecting loop to the α4 helix, but not its full height from the Mg^2+^ ions at the base to Y143 at the top as RAL and EVG do.

A distinguishing feature among the catalytic sites of the RAL-, EVG- and DTG-bound, IN-DNA models is the alternate conformations of the terminal 3′ adenylate (nucleotide A1) of the transferred DNA strand. Two conformations were modeled in the IN-DNA-RAL complex based on our analysis of the electron density of the PFV DNA in 3OYA (see Supplementary Experimental Procedures in File S1). One conformer has the adenine π-stacked against RAL’s metal-chelating scaffold (Figure 2A); the second has the adenine positioned near RAL’s 4-fluorobenzyl with the 2 in van der Waals contact (Figure 2B). In the IN-DNA-EVG model (Figure 2C), the adenine is in van der Waals contact with EVG’s 7-methyloxy group, which in turn positions the nucleobase away from the inhibitor’s 3-chloro-2-fluorobenzyl, creating a more open catalytic pocket compared with the adenylate conformation of the IN-DNA-RAL model in Figure 2B. For the IN-DNA-DTG model (Figure 2D), the adenine is π-stacked against DTG’s metal-chelating scaffold, where we presume that this nucleobase interacts favorably with the scaffold of the inhibitor through van der Waals forces.

Although many factors may directly or indirectly impact the conformation of the terminal 3′ adenylate, the structure of the IN inhibitor should have significant influence. In turn, the distinct conformations of this nucleotide will create different binding environments for the IN inhibitors. We speculate that the 3′ adenylate first pulls away from in between P145 and nucleotide C2 into a conformation resembling that of the IN-DNA-EVG model (Figure 2C). With this repositioning, there is also the concomitant loss of the coordinate bond between the adenylate’s 3′ hydroxyl group and the Mg^2+^ ion coordinated to residues D64 and E152, thus allowing an IN inhibitor to chelate both metal cations. Once the inhibitor is bound, the conformation of the 3′ adenylate may change depending on the inhibitor’s structure. The lone adenylate conformation in the EVG-bound, PFV intasomes, 3L2U and 3L2W [22], has the adenine interacting with EVG’s 7-methyloxy group (Figure 2C). We attribute this to EVG’s bulky 1-hydroxymethyl-2-methylpropyl group (Figure 1), which may effectively hinder the rotation of the adenine into a position where it stacks against EVG’s metal-chelating scaffold. The 2 alternate conformations that likely exist in the RAL-bound, PFV intasome, 3OYA, may be attributed to RAL’s smaller gem-dimethyl group, which may partially hinder the rotation of the adenine into a position where it stacks against the inhibitor’s metal-chelating scaffold. As for DTG, the lone conformation of the 3′ adenylate in the PFV intasome 3S3M, where the adenine is π-stacked against the inhibitor’s metal-chelating scaffold, is as expected since DTG does not possess a substituent para to the metal-chelating hydroxyl group that can hinder the rotation of the adenine.

**Figure 3:**
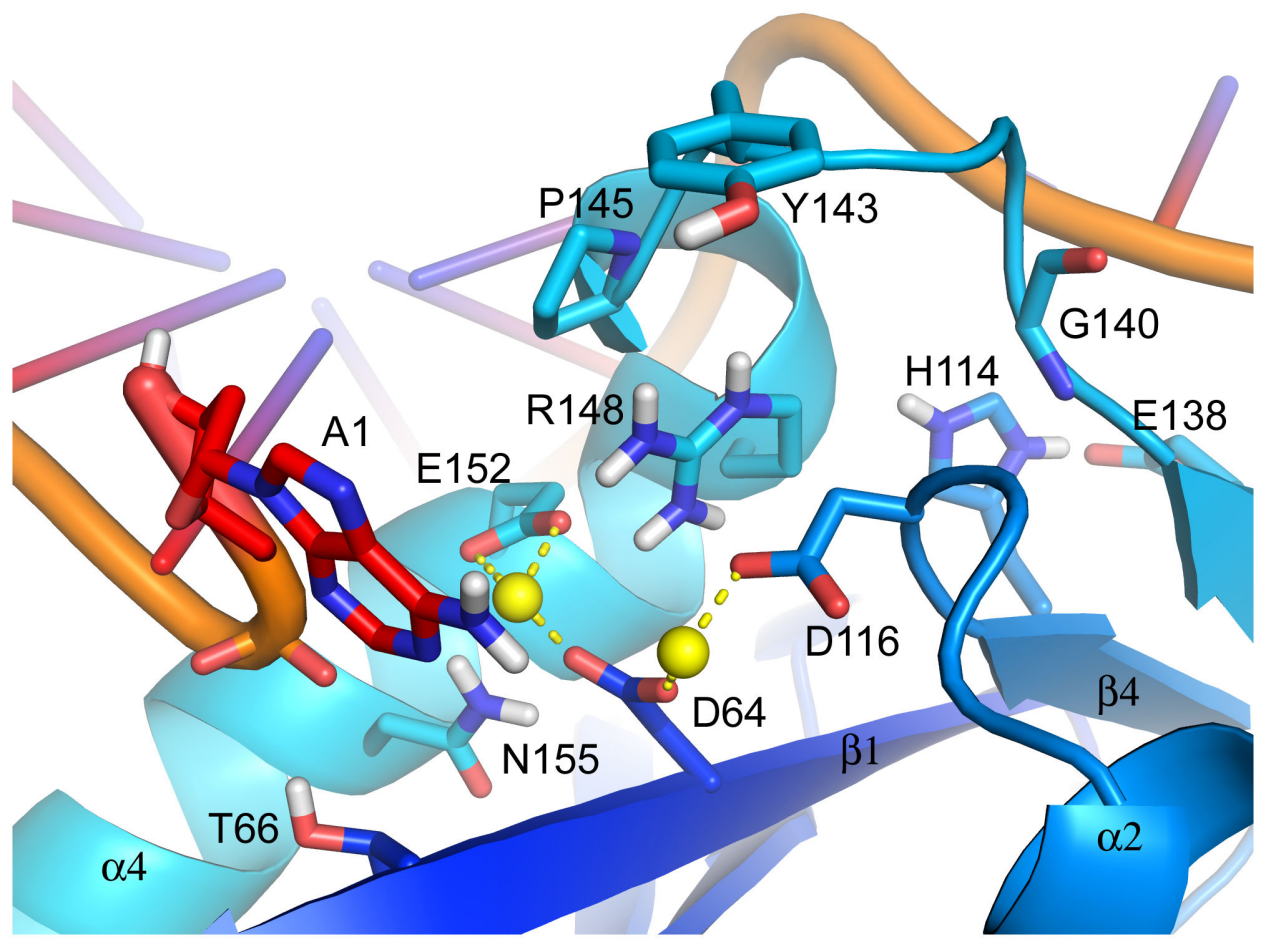
Structural model of Q148R HIV-1 integrase with U5 LTR DNA. The side chain of residue Q148R was modeled interacting with the side chain of E152 and in this conformation the residue may interfere with the binding of elvitegravir. Molecular representations and coloring schemes as described in Figure 2.

**Figure 4:**
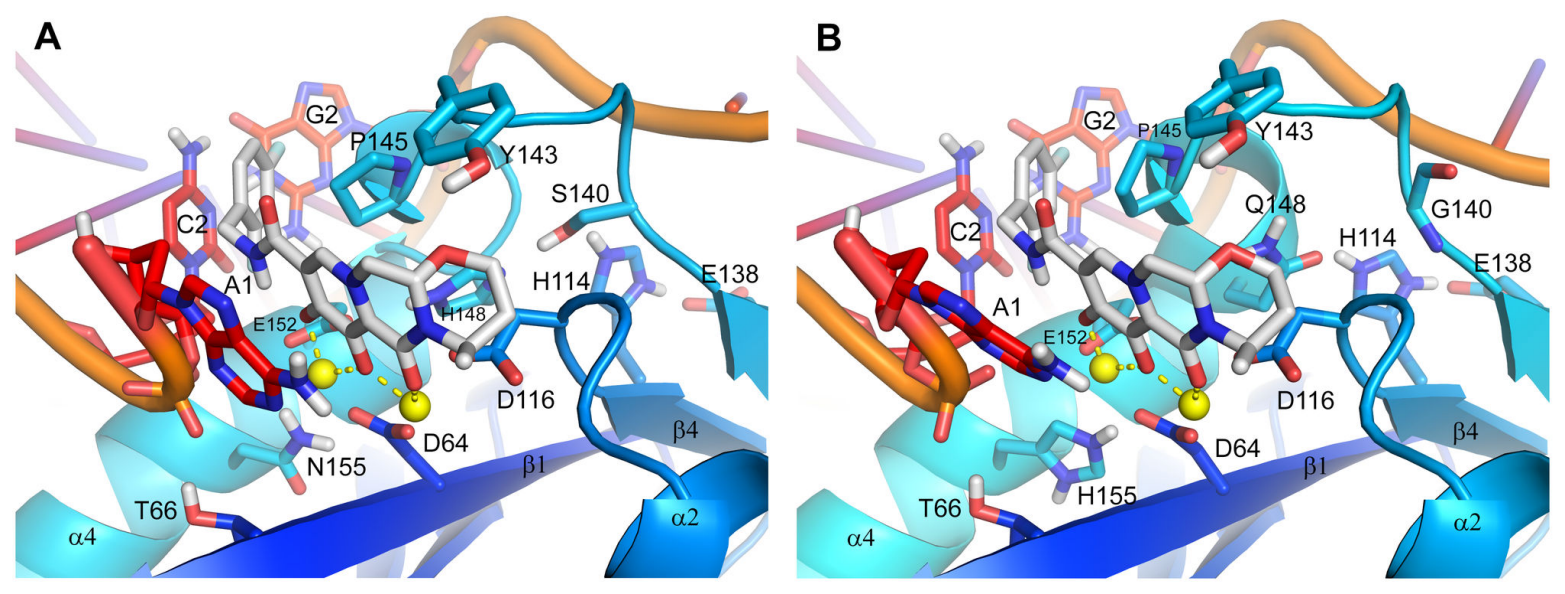
Structural models of (A) Q148H/G140S and (B) N155H HIV-1 integrase with U5 LTR DNA and dolutegravir. (A) The Q148H/G140S mutations are predicted to disrupt the structure of the flexible active-site loop, displacing the 3_10_ helix away from the DDE motif and weakening the H-bond interaction between the backbone CO of Q148H and the backbone NH of E152. (B) The N155H mutation is predicted to disrupt the structure of the α4 helix, widen the base of the catalytic pocket, alter the placement of at least the Mg^2+^ ion coordinated to residues D64 and E152 and alter the conformation of the terminal 3′ adenosine forming part of the pocket. Molecular representations and coloring schemes are described in Figure 2.

The catalytic sites of the Q148R, Q148H/G140S and N155H IN-DNA models are illustrated in Figures 3, 4A and 4B, respectively. The Q148R IN-DNA model does not include an IN inhibitor in its active site so as to highlight the ionic interaction between the side chains of Q148R and E152. A Q148K IN-DNA model was built, but as it reveals no new information, the model is not shown. The Q148H/G140S and N155H IN-DNA models have DTG docked into their active sites with poses set essentially identical to that of the IN-DNA-DTG model (Figure 2D). Whether liganded or not, the relevant differences among these mutant IN-DNA models actually lie in the structural disturbances induced by the residue substitutions.

For Q148R IN, residue P145 was displaced up and away from the base of the catalytic pocket, affecting residues Y143, N144 and Q146, but without disrupting the 3_10_ helix (comprised of residues N144-S147). For Q148H/G140S IN, the Q148H substitution perturbed the C-terminal end of the active-site loop displacing residues Y143 through G149 with the 3_10_ helix pushed away from the DDE motif. Residue Q148H, however, underwent the largest shift in position as evidenced by the roughly 1.0 Å displacement of its Cα atom relative to that of the wild-type residue, weakening the H-bond between its backbone CO and backbone NH of E152. It is difficult to discern the structural impact the G140S substitution may have on the active-site loop since Ser is the naturally occurring amino acid at PFV IN position 209, and a crystal structure of a S217Q/S209G PFV IN mutant is unavailable. Nevertheless, the structural effects of the G140S substitution are likely to be considerable given the obvious and significant difference between Gly and Ser. For N155H IN, the local structure of the α4 helix was distorted by the residue substitution, disrupting the H-bonds between the backbone CO of E152 and backbone NH of K156 and the backbone CO of S153 and backbone NH of E157 with distances of ≥3.5 Å between the heavy atoms. The N155H substitution also caused the base of the catalytic pocket to widen. For instance, the distance between the Cα atoms of D64 and N155H was increased by roughly 0.5 Å compared with wild-type IN. It may be argued that the placement of at least the Mg^2+^ ion coordinated to D64 and E152 is altered by the added steric bulk of His at N155, however, potential evidence for this from the PFV intasome structures lies at the limit of atomic position error.

A single conformation for the terminal 3′ adenylate of the Q148H/G140S and N155H IN-DNA complexes was modeled with the nucleobase π-stacked against DTG’s metal-chelating scaffold (Figures 4A and 4B, respectively). While the placement of these adenines is fairly similar to that of the IN-DNA-DTG model, the adenine from the N155H IN-DNA model is actually shifted up by roughly 0.5 Ǻ and angled away from DTG’s metal-chelating scaffold by almost 20° compared with the adenine from the IN-DNA-DTG model. In other words, the nucleobase is not parallel to the inhibitor’s scaffold creating a non-optimal π-stack between the adenine and DTG.

## Discussion

Many factors contribute to IN inhibitor efficacy and the development of resistance in vivo including residence time, drug pharmacokinetics, viral fitness and adherence. While it is not possible to replicate all of these components in the laboratory, studies conducted in vitro and in silico can provide insight into features that could contribute to IN inhibitor efficacy and impact the development of resistance. Here we discuss the structural rationale for the observed in vitro drug resistance and dissociation kinetics profiles of DTG, RAL and EVG based on our molecular models of the wild-type and mutant HIV-1 intasomes in complex with the aforementioned IN inhibitors. For the reader’s convenience, a subset of the relevant dissociation data from Hightower et al [10] along with the corresponding fold changes in in vitro anti-HIV activity (half-maximal effective concentration [EC_50_]) versus wild-type virus reported by Kobayashi et al [7] are provided in Table S1 in the File S1.

### Y143C/H/R HIV-1 Integrases

The π-stacking interaction between RAL’s 1,3,4-oxadiazole and Y143’s phenyl group in the IN-DNA-RAL model (Figures 2A-B) was first characterized by Hare et al through their RAL-bound, PFV intasome structures [22]. Clearly by mutating Y143 to Cys or Arg, the π-stacking interaction is eliminated thus negatively affecting binding. Although the side chain of His is aromatic, mutating Y143 to His should also compromise binding given the possible tautomeric and protomeric states of His, the necessary alignment of electronic charge distributions between residue and inhibitor, and their diminished polarization and dispersion interactions. The 4.4-, 3.5- and 8-fold decreases in RAL’s dissociative t_1/2_ caused by the Y143C/H/R mutations (Table S1 in File S1), respectively, provide evidence for the compromised inhibitor binding to the mutant enzymes. While there is no direct correlation between in vitro IN inhibitor dissociation and antiviral potency, and consequently in vitro resistance, it has been reported that mutations that result in fast IN inhibitor dissociation also tend to cause significant decreases in in vitro antiviral potency [10,33]. The Y143C/R mutations confer significant resistance to RAL as supported by the 3.2- and 16-fold increases in EC_50_, respectively, and we speculate that these results are likely in part due to RAL’s faster off-rates from these IN mutants. Although the Y143H mutation is not associated with a substantial loss in anti-HIV activity, the minor 1.8-fold increase in EC_50_ is likely associated with the decreased dissociative t_1/2_ of RAL. Notably, the Y143R mutation had the most negative effect on RAL’s antiviral activity consistent with the magnitude of the decrease in its dissociative t_1/2_, both of which were expected given the size, flexibility and formal charge of Arg.

The IN-DNA models in complex with EVG (Figure 2C) and DTG (Figure 2D) show that both inhibitors make limited van der Waals contact with the side chain of Y143, EVG through its isopropyl group and DTG through its tetrahydro-oxazine group. This likely explains why all 3 Y143 mutations have only modest effects on their dissociative t_1/2_ with decreases in value relative to wild-type IN ranging from 1.2- to 1.7-fold. Consistent with these results, the in vitro anti-HIV activities of EVG and DTG remain at or near wild-type level against the viral mutants harboring the Y143 mutations. Note, from Table S1 in File S1, however, that EVG’s dissociative t_1/2_ values with Y143C/H/R, which range from 1.6 to 2.1 hours, are comparable in magnitude to those of RAL, yet EVG has near wild-type antiviral activity. We attribute this to EVG’s carboxylate, which coordinates the pair of Mg^2+^ ions at the catalytic site. Consider that the hydroxyl group of RAL, as well as that of DTG, must first be deprotonated in order to form coordinate bonds with the metal cations, which themselves facilitate deprotonation by lowering the associated free energy. In contrast, EVG’s carboxylic acid is predominantly deprotonated in solution, and as such, the inhibitor’s on-rate should be considerably faster than those of RAL and DTG, counterbalancing EVG’s faster off-rate. Dolutegravir maintains prolonged binding with the Y143C/H/R mutants as demonstrated by dissociative t_1/2_ values of 42 to 60 hours. In addition to its size and electronic properties as discussed later, we speculate that the streamlined architecture of DTG’s metal-chelating scaffold is another contributing factor to the compound’s prolonged binding and antiviral activity as it can potentially π-stack against the terminal 3′ adenine, which may hinder the egress of DTG from the active site.

The IN-DNA-RAL model in Figure 2A shows a conformation of the 3′ adenylate that has the nucleobase π-stacked against RAL’s metal-chelating scaffold similar to what is seen in 3OYA. From this, the expectation is that RAL’s off-rate and antiviral activity should be positively affected similarly to DTG. However, the IN-DNA-RAL model also includes an alternate adenylate conformation with its nucleobase lying near RAL’s 4-fluorobenzyl as illustrated in Figure 2B. This conformation is consistent with our analysis of the 3OYA electron density, which suggested that a second nucleotide conformation may be present in approximately equal proportion to that shown in Figure 2A. With the adenine positioned away from RAL’s metal-chelating scaffold, the adenine has more limited van der Waals contact with the inhibitor and thereby a less stabilizing influence. Moreover, the nucleotide may not hinder the egress of RAL from the active site as effectively when in the alternate conformation shown in Figure 2B. Therefore, the potential for prolonging RAL’s binding to HIV-1 IN in the same way as DTG is less likely.

### Q148H/K/R HIV-1 Integrases

Residue Q148 is located at the C-terminal end of the active-site loop between the 3_10_ helix that forms part of the loop and the α4 helix. In the wild-type IN model (Figure 2) the side chain of Q148 forms an H-bond with the side chain of H114, which was modeled positively ionized since it also interacts with the side chain of E138. We speculate that this H-bond network may help stabilize the C-terminal end of the active-site loop with residue G140 imparting flexibility at the N-terminal end limited only by a potential H-bond between its backbone NH and the backbone CO of N115. A comparison of the wild-type and Q148H/G140S IN models suggests that the Q148H mutation alone may displace residues Y143 through G149 from their wild-type positions because of the larger steric bulk of His versus Asn and the plausible redirection of the H-bond trajectory between the side chains of Q148H and H114 assuming the interaction forms. For Q148H IN, we predict that the mutant residue still undergoes a large positional shift away from the DDE motif, but without disruption of the 3_10_ helix, and consequently still creates strain at the C-terminal end of the active-site loop weakening the H-bond between the backbone CO of Q148H and backbone NH of E152 as in the Q148H/G140S IN model.

While we recognize that the structural disturbances induced by the Q148H/G140S mutations are not solely attributable to residue Q148H, it is possible that these changes are simply not as pronounced in Q148H IN given that it merely has to accommodate the “hydrogen” side chain of G140, whereas Q148H/G140S IN has the hydroxymethyl side chain of G140S. However, it is also possible that the N-terminal end of Q148H IN’s active-site loop may become more disordered because of the flexibility that residue G140 imparts on the loop, the inability of residue G140 to H-bond with the side chains of Q148H and E138, and the strain energy induced by the mutation itself. Biochemical support for the increased disorder of the active-site loop of Q148H IN lies in the fact that the catalytic activity of the mutant enzyme is severely impaired [34].

Similar to Q148H IN, we predict that the Q148K/R mutations also perturb the structure of the active-site loop given the larger steric bulk of Lys and Arg versus Asn and the steric clash with residue P145 that existed prior to structural refinement of the Q148R/K IN models. Following geometry optimization, residue P145 was found to be displaced from its wild-type position, up and away from the base of the catalytic pocket affecting the positions of neighboring residues Y143, N144 and Q146, but without disruption of the 3_10_ helix (Figure 3). Although not clear from the Q148R/K models, we nonetheless expect the H-bond between the backbone CO of Q148H and backbone NH of E152 to be weakened because of the strain induced by the mutations. With residue G140 at the N-terminal end of the active-site loop, the expectation again is that the loop becomes more disordered as supported biochemically by the severely impaired catalytic activities of the Q148K/R IN mutants [34].

Along with the predicted structural disturbances that the Q148H/K/R mutations may induce, there exist the inherent associated strain energies. Consequently, the binding of RAL, EVG and DTG should be negatively impacted as the enzyme adjusts to mitigate the structural strain. This is supported experimentally by the shortened dissociative half-lives of the inhibitors relative to wild-type IN (Table S1 in File S1). The Q148H mutation reduced the dissociative t_1/2_ of RAL by 44-fold to 0.2 hours, whereas those of DTG and EVG were reduced roughly 13.5-fold to 5.2 and 0.2 hours, respectively. The decreases in RAL’s dissociative t_1/2_ produced by the Q148K/R mutations were ≥22-fold to 0.3 and 0.4 hours, respectively, which were greater than those of DTG with decreases of 6.5- to 7.7-fold to 11 and 9.2 hours, respectively. For EVG, there was insufficient binding to the IN-DNA complex to measure a dissociative t_1/2_ involving Q148K/R IN. Where comparisons are possible, note the greater negative impact of the Q148H/K/R IN mutations on RAL’s binding compared with DTG and EVG. We attribute this to the potential displacement of residue Y143 that may arise from the structural disturbances predicted for the active-site loop, and the subsequent compromise in the π-stacking interaction between RAL’s 1,3,4-oxadizaole and Y143’s phenyl group.

The losses in anti-HIV activity for RAL of 13-, 83- and 47-fold against the Q148H/K/R IN viral strains, respectively, likely reflect the decreases in binding to the mutant IN-DNA complexes. However, the losses in potency against the Q148K/R IN viral strains are far greater than expected when one considers the inhibitor’s fold increase in EC_50_ for the Q148H IN mutant against the shortened dissociative t_1/2_. To account for this, we speculate that the predicted displacement of residue P145 by the Q148K/R mutations may negatively affect RAL’s on-rate as the side chain of P145 may lie in position to sterically hinder the inhibitor’s approach through its gem-dimethyl group.

For EVG, the reduced anti-HIV activity against the Q148H/K/R IN viral strains is consistent with decreased binding to the mutant IN-DNA complexes. However, greater losses in antiviral potency were observed for the Q148K/R IN viral strains with fold increases in EC_50_ of >1700 and 240, respectively, versus the 7.3-fold increase against the Q148H IN mutant. We propose based on the predicted rotameric state of Arg in Q148R IN (Figure 3) and Lys in Q148K IN (not shown) that EVG’s carboxylate will preferentially form an ionic interaction with the positively charged side chains of these residues, consequently impeding EVG from chelating the metal cations at the catalytic site. This hypothesis may help to explain why insufficient signal was generated for EVG with the IN-DNA complexes containing Q148K/R substitutions in the dissociation studies [10].

In contrast to RAL and EVG, the Q148H/K/R IN mutations had no appreciable effect on the in vitro anti-HIV activity of DTG, despite the inhibitor’s faster dissociation compared with wild-type IN. While DTG’s binding is reduced by the IN mutations, its dissociative half-lives are still fairly substantial, ranging in value from 5.2 to 11 hours, which are roughly comparable to that of RAL with the wild-type IN-DNA complex at 8.8 hours. Here again, we speculate that an important contributing factor to DTG’s prolonged binding is its streamlined, metal-chelating scaffold and its potential to π-stack against the terminal 3′ adenine (Figure 2D). We predict that this π-stacking interaction will not be appreciably affected by the Q148H/K/R mutations given the residue’s position within the catalytic pocket. Moreover, as DTG’s scaffold does not occupy the pocket’s full height, the potential displacement of residues about the midpoint of the active-site loop, like Y143, should have minimal effects on DTG’s dissociation rate and antiviral activity.

### Q148H/G140S HIV-1 Integrase

Q148H/G140S is the most common combination of primary and secondary IN mutations within the Q148 resistance pathway, likely due to the almost full restoration of catalytic activity with the addition of G140S to Q148H [34]. We attribute this to the formation of an H-bond network between residues H114, E138, G140S and Q148H, which may help stabilize the active-site loop into a near wild-type, catalytically active conformational state decreasing the disorder of the loop introduced by the primary mutation. The Q148H/G140S IN-DNA model (Figure 4A) shows, however, that the amino acid substitutions do affect the structure of the active-site loop displacing most of its residues from their wild-type positions; the side chains of H114 and E138 are also displaced to accommodate the G140S mutation and form some of the H-bonds in the specified network. Residue Q148H undergoes the largest positional shift away from the DDE motif in order to accommodate the larger steric bulk of His versus Gln creating strain in the enzyme and weakening the H-bond between the backbone CO of Q148H and backbone NH of E152.

Because of the structural disturbances and associated strain energy arising from the Q148H/G140S mutations, the binding of RAL, EVG and DTG should be negatively impacted; this is clearly supported by their shortened dissociative half-lives relative to wild-type IN (Table S1 in File S1). For RAL, its dissociative t_1/2_ was decreased 44-fold to 0.2 hours, but for EVG there was insufficient binding to measure a value. Raltegravir’s antiviral activity was decreased >130-fold, which is >10-fold over that reported for Q148H IN reflecting the further decrease in binding that we attribute to the decrease in flexibility of the active-site loop and the inhibitor occupying the catalytic pocket’s full height. With the active-site loop constrained by a network of H-bonds, the on-rate of RAL may slow as the compound moves to occupy the catalytic pocket. For EVG, the decrease in antiviral activity was >890-fold, which we speculate reflects the structural strain induced by the Q148H/G140S mutations. Dolutegravir’s dissociative t_1/2_ was decreased 21.5-fold to 3.3 hours; this is associated with a 2.6-fold decrease in antiviral activity, which is near the 3-fold mark that is considered resistant [7].

### N155H HIV-1 Integrase

Residue N155 forms part of the α4 helix near its N-terminal end with the residue’s side chain directed towards the β1 strand. From this position, the N155H mutation may induce the structural disturbances modeled in the N155H IN-DNA complex (Figure 4B), which include deformation of the α4 helix through disruption of backbone H-bonds among the neighboring residues, E152, S153, K156 and E157, and a widening of the catalytic pocket’s base between residues D64 and N155H to accommodate the larger steric bulk of His versus Asn. These structural changes along with the associated strain energies should negatively impact the binding of RAL, EVG and DTG as the enzyme adjusts to mitigate the structural strain imposed by the IN mutation. This hypothesis is supported by the shortened dissociative half-lives of all 3 inhibitors. For RAL, its dissociative t_1/2_ was reduced 14.7-fold to 0.6 hours, whereas for EVG, it was reduced 6.8-fold to 0.4 hours, and the loss of anti-HIV activity of 8.4-fold for RAL and 25-fold for EVG likely reflect their decrease in binding (Table S1 in File S1). Although DTG’s dissociative half-life was decreased 7.4-fold, the inhibitor still exhibits prolonged binding as demonstrated by its t_1/2_ of 9.6 hours and is thus reflected in its wild-type activity against the viral mutant.

The N155H mutation had a greater negative effect on RAL’s dissociative t_1/2_ compared with those of EVG and DTG. We speculate that RAL’s faster dissociation may result from a potential shift of the coordination complex within the catalytic pocket that is attributable to the larger steric bulk of His. Support from the N224H PFV intasome structures in 3OYN [23] and 3S3O is not definitive since the positional shifts of the Mg^2+^ ions (in particular, that coordinated to D128 and E221), and non-hydrogen atoms of the complexing ligands (eg, residues of the DDE motif, DTG) are at or near the boundary of atomic position error. Nevertheless, even a slight shift in the coordination complex can compromise the π-stacking interaction between RAL’s 1,3,4-oxadiazole and Y143’s phenyl group, thus more substantially reducing RAL’s binding compared with DTG and EVG, which make only limited van der Waals contact with residue Y143.

Dolutegravir’s slow dissociation from the N155H IN-DNA complex has similar underpinnings as described for the Q148H/K/R mutations. The lack of a substituent para to DTG’s hydroxyl group, we predict, allows the terminal 3′ adenine to π-stack against the inhibitor’s scaffold as shown in the N155H IN-DNA-DTG model (Figure 4B). Although this π-stacking interaction is non-optimal due likely to the N155H mutation, the nucleobase is nonetheless in position to also slow the egress of DTG from the catalytic pocket.

### Structural and Electronic Properties of DTG, RAL and EVG

To understand the in vitro resistance and dissociation kinetics profiles of RAL, EVG and DTG more fully, the structural and electronic properties of the IN inhibitors must also be considered. One structural feature of DTG already discussed is the streamlined architecture of its metal-chelating scaffold with its potential to π-stack against the terminal 3′ adenine thus further increasing inhibitor binding. Compared with RAL, DTG also has a larger metal-chelating scaffold (Figure 1) and should therefore form stronger coordinate bonds to the metal cations given that its scaffold should more effectively delocalize the build of positive charge on the ligating hetero-atoms as these bonds form. Although EVG may appear to have a sizable metal-chelating scaffold, the delocalization of charge between its carboxylate and quinolinone should be significantly hampered by the fact that these functional groups will be out-of-plane from each other. Since EVG will be mostly deprotonated in solution, its on-rate should be faster than those of DTG and RAL, however, its limited capacity to delocalize charge means that its off-rate is likely faster too. Although the electronic properties just cited can be investigated and verified quantum mechanically, such work is beyond the scope of the present study.

In efforts to explain the structural basis for DTG’s potency against RAL-resistant IN enzymes, Hare et al reported that the length and flexibility of DTG’s N-methylamide linker allows the compound’s difluorophenyl group to penetrate farther into the pocket vacated by the terminal 3′ adenine thus forming a more favorable π-stacking interaction with the base of nucleotide C2 compared with the halophenyl groups of RAL, EVG and MK-2048 [24]. It is important to note, however, that these phenyl groups of RAL, EVG, MK-2048 and DTG are substituted differently with halogens and as a result may not superimpose thus accounting to some extent for their displacements as seen crystallographically. As for the flexibility of DTG’s N-methylamide linker, Hare et al reported that the π-conjugation about the amide and metal-chelating core restricts the rotation of the amide bond and the bond connecting these 2 groups, and we agree. However, also note that DTG’s amide NH and 8-oxo group form an intramolecular H-bond creating a pseudo 6-member ring. Together the π-conjugation and H-bonding should effectively reduce the number of freely rotating bonds between the phenyl and metal-chelating core to 2. It can be argued instead that RAL’s N-methylamide linker has a greater degree of flexibility as its amide NH and pyrimidinyl N^3^ atom form a pseudo 5-member ring creating a relatively weaker intramolecular H-bond. Our expectations are consistent with these inhibitors’ potencies and the theory that a more flexible compound should have a less favorable binding free energy compared with a less flexible one due to the greater loss of conformational entropy upon binding.

In this work, molecular modeling was used to provide new insights into the distinct in vitro drug resistance and dissociation profiles of RAL, EVG and DTG with regard to RAL signature mutations. For DTG, its streamlined architecture and its theorized electronic properties are potential key factors which differently influence the catalytic pocket and metal coordination and impact both retention of antiviral potency and prolonged inhibitor binding against wild-type and mutant IN. The characteristics of DTG elucidated through this study also provide insight into the clinical efficacy that has been observed with wild-type and most RAL- and EVG-resistant viruses such as with Y143 and N155 pathway genotypes, and for DTG’s decreasing efficacy against Q148 pathway viruses with increasing numbers of secondary mutations[8]. This modeling study in conjunction with antiviral, biochemical and clinical evidence highlights the potential for DTG to possess a higher barrier to resistance in vivo.

## Supporting Information

Figure S1 **(**A**) MVP-calculated sequence alignment of the NL432 HIV-1 [1] and PFV [2] IN amino acids (GenBank: AAC61700.1 and PDB: 3L2Q_A, respectively).** The 1-letter codes for the residues are color coded: Ala, Val, Leu, Ile, Met, Phe, Tyr and Trp are in green; Lys and Arg are in blue; His is in teal; Glu and Asp are in red; Ser, Thr and Cys are in brown; Asn and Gln are in purple; and Pro and Gly are in black. The yellow boxes outlined in red capture those amino acids in α or 3_10_ helices (captions starting with “a” and “h”, respectively); the red boxes outlined in blue capture those amino acids in β-strands (caption starting with “b”); and the small boxes outlined in black highlight the residues of the DDE motif. The secondary structural elements are numbered for the individual integrase enzyme domains. The 3_10_ helix labeled “h2” is only present in the PFV IN catalytic core domain. (B) Nucleotide sequence used to model the HIV-1 U5 LTR end. The 2 nucleotides highlighted in yellow are not part of the 3′ processed DNA model.
(DOCX)

File S1 **Construction and refinement of molecular models. **
(DOCX)
