# Supplementary figures and images for: Dolutegravir Interactions with HIV-1 Integrase-DNA: Structural Rationale for Drug Resistance and Dissociation Kinetics
Source: PLoS One. 2013 Oct 16;8(10):e77448. doi: 10.1371/journal.pone.0077448 (PMC3797783; doi:10.1371/journal.pone.0077448)

**A**


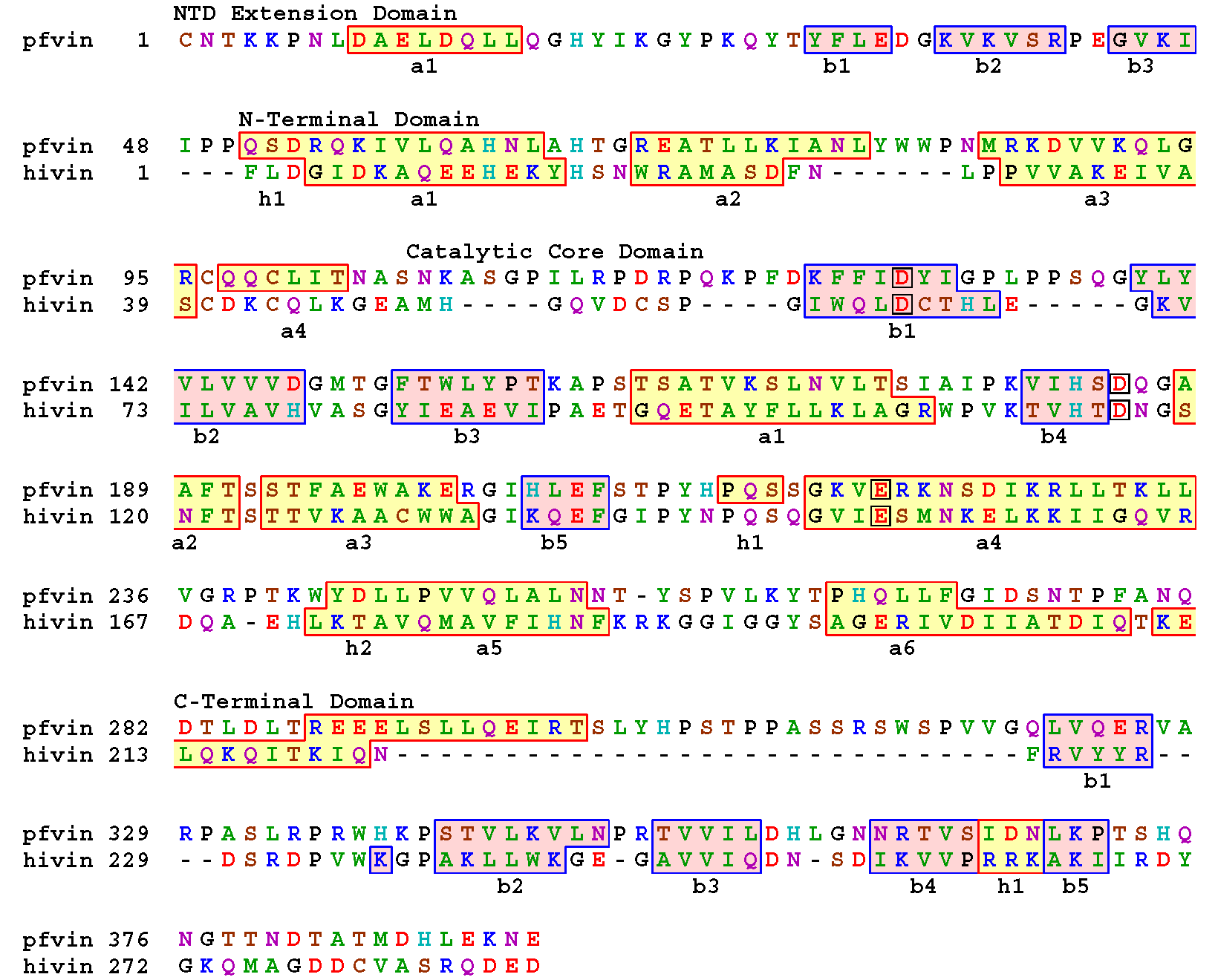


**B**

| **17** | **16** | **15** | **14** | **13** | **12** | **11** | **10** | **9** | **8** | **7** | **6** | **5** | **4** | **3** | **2** | **1** | **-1** | **-2** |  |
| --- | --- | --- | --- | --- | --- | --- | --- | --- | --- | --- | --- | --- | --- | --- | --- | --- | --- | --- | --- |
| G | T | G | G | A | A | A | A | T | C | T | C | T | A | G | C | A | G | T | 3′ |
| C | A | C | C | T | T | T | T | A | G | A | G | A | T | C | G | T | C | A | 5′ |

Supplement: Figure S1 — (A) MVP-calculated sequence alignment of the NL432 HIV-1 [1] and PFV [2] IN amino acids (GenBank: AAC61700.1 and PDB: 3L2Q_A, respectively). The 1-letter codes for the residues are color coded: Ala, Val, Leu, Ile, Met, Phe, Tyr and Trp are in green; Lys and Arg are in blue; His is in teal; Glu and Asp are in red; Ser, Thr and Cys are in brown; Asn and Gln are in purple; and Pro and Gly are in black. The yellow boxes outlined in red capture those amino acids in α or 310 helices (captions starting with “a” and “h”, respectively); the red boxes outlined in blue capture those amino acids in β-strands (caption starting with “b”); and the small boxes outlined in black highlight the residues of the DDE motif. The secondary structural elements are numbered for the individual integrase enzyme domains. The 310 helix labeled “h2” is only present in the PFV IN catalytic core domain. (B) Nucleotide sequence used to model the HIV-1 U5 LTR end. The 2 nucleotides highlighted in yellow are not part of the 3′ processed DNA model. (DOCX) [file pone.0077448.s001.docx]
