# Supplementary material for: Dolutegravir Interactions with HIV-1 Integrase-DNA: Structural Rationale for Drug Resistance and Dissociation Kinetics
Source: PLoS One. 2013 Oct 16;8(10):e77448. doi: 10.1371/journal.pone.0077448 (PMC3797783; doi:10.1371/journal.pone.0077448)
Supplement: File S1 — Construction and refinement of molecular models. (DOCX) [file pone.0077448.s002.docx]

**Dolutegravir Interactions With HIV-1 Integrase-DNA: Structural Rationale for Drug Resistance and Dissociation Kinetics**

Felix DeAnda,^1^ Kendra E. Hightower,^1^ Robert T. Nolte,^1^ Kazunari Hattori,^2^ Tomokazu Yoshinaga,^2^ Takashi Kawasuji,^2^ Mark R. Underwood^1^

^1^GlaxoSmithKline, Research Triangle Park, NC, USA; ^2^Shionogi & Co., Ltd., Osaka, Japan

**SUPPLEMENTAL DATA**

**(A)**
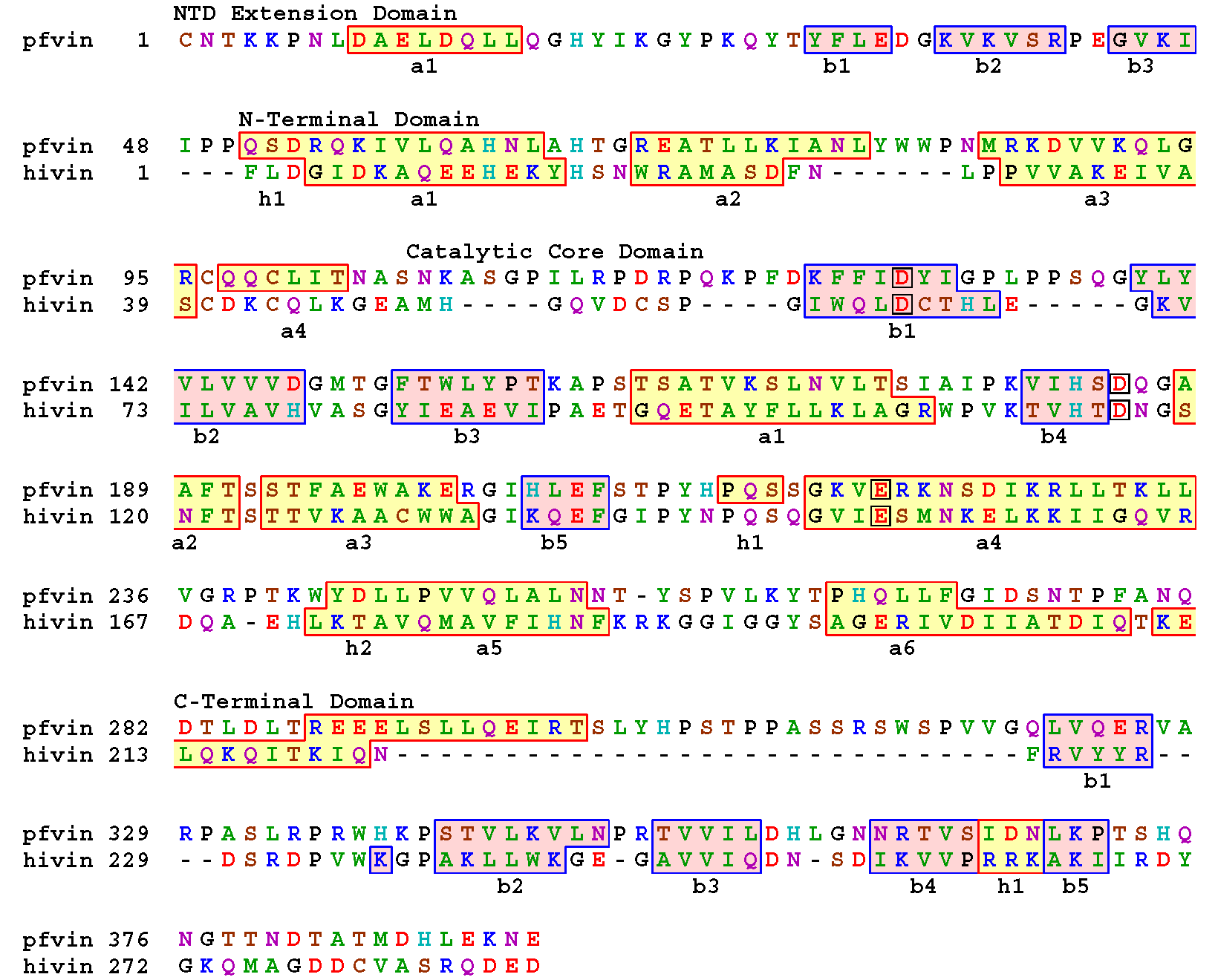


**(B)**

| **17** | **16** | **15** | **14** | **13** | **12** | **11** | **10** | **9** | **8** | **7** | **6** | **5** | **4** | **3** | **2** | **1** | **-1** | **-2** |  |
| --- | --- | --- | --- | --- | --- | --- | --- | --- | --- | --- | --- | --- | --- | --- | --- | --- | --- | --- | --- |
| G | T | G | G | A | A | A | A | T | C | T | C | T | A | G | C | A | G | T | 3′ |
| C | A | C | C | T | T | T | T | A | G | A | G | A | T | C | G | T | C | A | 5′ |

**Figure S1.** **(A)** MVP-calculated sequence alignment of the NL432 HIV-1 [[1](#_ENREF_1)] and PFV [[2](#_ENREF_2)] IN amino acids (GenBank: AAC61700.1 and PDB: 3L2Q_A, respectively). The 1-letter codes for the residues are color coded: Ala, Val, Leu, Ile, Met, Phe, Tyr, and Trp are in green; Lys and Arg are in blue; His is in teal; Glu and Asp are in red; Ser, Thr, and Cys are in brown; Asn and Gln are in purple; and Pro and Gly are in black. The yellow boxes outlined in red capture those amino acids in α or 3_10_ helices (captions starting with “a” and “h,” respectively); the red boxes outlined in blue capture those amino acids in β-strands (caption starting with “b”); and the small boxes outlined in black highlight the residues of the DDE motif. The secondary structural elements are numbered for the individual integrase enzyme domains. The 3_10_ helix labeled “h2” is only present in the PFV IN catalytic core domain. **(B)** Nucleotide sequence used to model the HIV-1 U5 LTR end. The 2 nucleotides highlighted in yellow are not part of the 3′ processed DNA model.**Table S1.** Reference Data From HIV-1 IN Inhibitor In Vitro Dissociation and Antiviral Activity Studies

|  | **Dissociative t_1/2_ (h)^a^** | | |  | | **Fold change in EC_50_^b^** | | | |
| --- | --- | --- | --- | --- | --- | --- | --- | --- | --- |
| **IN** | **DTG** | **RAL** | **EVG** | |  | | **DTG** | **RAL** | **EVG** |
| Wild-type | 71 | 8.8 | 2.7 | |  | | 1 | 1 | 1 |
| Y143C | 60 | 2.0 | 2.1 | |  | | 0.95 | 3.2 | 1.5 |
| Y143H | 44 | 2.5 | 1.6 | |  | | 0.89 | 1.8 | 1.5 |
| Y143R | 42 | 1.1 | 1.7 | |  | | 1.4 | 16 | 1.8 |
| Q148H | 5.2 | 0.2 | 0.2 | |  | | 0.97 | 13 | 7.3 |
| Q148K | 11 | 0.3 | ND | |  | | 1.1 | 83 | >1700 |
| Q148R | 9.2 | 0.4 | ND | |  | | 1.2 | 47 | 240 |
| N155H | 9.6 | 0.6 | 0.4 | |  | | 0.99 | 8.4 | 25 |
| Q148H/G140S | 3.3 | 0.2 | ND | |  | | 2.6 | >130 | >890 |

^a^ Dissociative t_1/2_ values previously reported by Hightower et al, 2011 [[3](#_ENREF_3)]. ND denotes not determined because of insufficient signal in the assay.

^b^ Fold change in half-maximal effective concentration (EC_50_) versus wild-type HIV-1. Viral strains with a fold change ≥3 were considered resistant. Data previously reported by Kobayashi et al, 2011 [[4](#_ENREF_4)].

**SUPPLEMENTAL EXPERIMENTAL PROCEDURES**

Structural models of the dimeric catalytic core of wild-type, Q148H/G140S and N155H HIV-1 integrase (IN) were constructed starting from the HIV-1 IN structure in the RCSB Protein Data Bank (PDB; www.pdb.org) [[5](#_ENREF_5)] entry, 2B4J [[6](#_ENREF_6)]. Their flexible active-site loops, portions of their α4 helices, and the Mg^2+^-bound conformations of their acidic residues of the DDE motif were modeled from the corresponding residues in the wild-type, S217H and N224H prototype foamy virus (PFV) intasome structures from PDB entries 3OYA, 3S3N and 3S3O [[2](#_ENREF_2),[7](#_ENREF_7),[8](#_ENREF_8)], respectively. A Q148R and Q148K HIV-1 IN model were also built by mutating the pertinent residue from a copy of the wild-type IN model to Arg and Lys, respectively, and then setting the rotameric state of their side chains to match that of the equivalent Tn5 transposase residue, R322, from PDB entry 1MUS [[9](#_ENREF_9)]. Next, HIV-1 IN-DNA complexes were assembled by combining the wild-type and mutant IN models with distinct models of the HIV-1 U5 long terminal repeat (LTR) end, which were constructed from select PFV DNA templates that were chosen based on the IN inhibitor to be docked at the catalytic site given our speculation that the compound itself may affect the conformation of the terminal 3′ adenylate. For the wild-type IN-DNA complex, dolutegravir (DTG), raltegravir (RAL) and elvitegravir (EVG) were docked into its catalytic site guided by their binding modes in the PFV intasome, whereas only DTG was docked into the catalytic sites of the Q148H/G140S and N155H HIV-1 IN-DNA complexes, here again guided by its binding mode in the S217H and N224H PFV intasomes, respectively. No inhibitors were docked into the Q148R and Q148K IN-DNA models to purposely highlight the ionic interactions of the mutant residues.

As a first step in building our models, the pairwise sequence alignment of the NL432 HIV-1 [[1](#_ENREF_1)] and PFV [[2](#_ENREF_2)] IN amino acids (GenBank: AAC61700.1 and PDB: 3L2Q_A, respectively) was calculated using the Molecular Viewing Program (MVP) [[10](#_ENREF_10)]. The sequence alignment is illustrated in Figure 1A (related to Figure 2) and was found to be nearly identical to the structure-based sequence alignment reported by Hare et al [[2](#_ENREF_2)]. Based on the MVP alignment, the sequence identity between the common domains of HIV-1 and PFV IN was calculated to be roughly 19%. Despite the low sequence homology, there is still intuitive appeal to using PFV IN (or portions thereof) as a structural template for HIV-1 IN (or portions thereof) given that these retroviral enzymes are structurally and mechanistically related. The superimposition of their common catalytic cores reveals that these domains share a very similar RNase H-like fold even though their sequence identity is only roughly 21%. Focusing on their catalytic sites, which we define as those residues within 8.0 Å of RAL, EVG or DTG in PFV IN and their HIV-1 IN counterparts, the sequence identity is significantly higher at roughly 45%. The similar architecture shared by the 2 catalytic sites draws attention to the fact that the 1 essential missing component from chain A of the HIV-1 IN dimer in 2B4J is the active-site loop. The sequence identity between the HIV-1 and PFV IN active-site loops (residues G140-G149 and S209-G218, respectively) is 60%, leading us to speculate that perhaps their conformations resemble or are nearly identical to each other when in a catalytically active and inhibitor-bound state. However, there are 2 key residue differences between these loops located at or near their N- and C-terminal ends, which may lead to differences in flexibility and overall structure; these are HIV-1 IN residues G140 and Q148 and the corresponding PFV IN residues S209 and S217, respectively. With this in mind, we nonetheless chose to use the active-site loop of PFV IN as a model template for that missing from chain A of the HIV-1 IN dimer.

Although all published PFV IN structures were carefully considered, the active-site loop of chain A from the PFV IN dimer in 3OYA was selected as the template for the wild-type HIV-1 IN model. To model the Mg^2+^-bound rotameric state of HIV-1 IN residue E152, we chose to extend the PFV IN template by 3 amino acids to include K219 through E221, thus adding approximately 1 helical turn of the α4 helix to the template. For the Q148H/G140S HIV-1 IN model, the active-site loop of chain A from the S217H PFV IN dimer in 3S3N was selected as the template to model the structural disturbances that the 2 HIV-1 IN mutations may induce. Similar to the wild-type IN model, we extended the PFV IN template by residues K219 through E221 to once again model the metal-bound conformation of HIV-1 IN residue E152. For the N155H HIV-1 IN model, the active-site loop from chain A of the N224H PFV IN dimer in 3S3O was selected as the template. Unlike the other 2 IN models, however, we chose to extend the PFV IN template by 10 amino acids from K219 through K228 to model not only the metal-bound conformation of HIV-1 IN residue E152, but also the significant structural disturbances that the N155H mutation may induce on the α4 helix and surrounding environment.

With the loop templates selected, we next collected the structure factors for 3OYA, 3S3N and 3S3O from the PDB and proceeded with an analysis of the electron density maps in an effort to glean additional structural information. The EVG- and DTG-bound, PFV intasomes from PDB entries 3L2U and 3S3M, respectively, were also included in this exercise as these structures were chosen to serve as templates in the assembly of HIV-1 IN-DNA models in complex with EVG and DTG. Electron density maps were calculated for each PFV-related structure by performing a standard round of refinement with the program phenix.refine [[11](#_ENREF_11)] using program defaults. A second round of omit maps was similarly calculated where the terminal 3′ adenylate and ligand were removed. Where the omit map did not unambiguously match the adenylate and ligand positions, these were rebuilt into the omit map and carried through a third round of refinement to generate another set of maps, which were then compared with the first. Where we judged the final density fit to be better for our modifications of the above PFV-related structures, we chose to use the modified positions for our HIV-1 structural models.

Overall, the electron density maps were consistent with the published structures with a few exceptions. For 3OYA, there was only 1 conformation published for the terminal 3′ adenylate of PFV DNA. However, we found that the electron density was far more consistent with the adenylate in 2 alternate conformations of approximately equal occupancies. For 3S3N, the electron density appeared to support 2 alternate 3′ adenylate conformations with roughly equal weighting as published. However, the conformer with the adenine located near PFV nucleotide C16 was drawn into question given the distance of ~2.8 Å between the H-bond acceptors, N7 of the 3′ adenylate and O3 of PFV nucleotide C15. We supposed that an intervening ion might be present to coordinate the interaction between the H-bond acceptors, but this proved difficult to support crystallographically. We also supposed that a small molecule and not the adenylate was present there, but this also proved difficult to support. In the end, this adenylate conformer was dismissed. For the conformer with the adenine π-stacked against DTG’s metal-chelating scaffold, we found that the density was more consistent with the reported adenylate conformations in the DTG-bound, PFV intasomes in 3S3M and 3S3O. Based on our observations, we speculated that the adenine may primarily interact with DTG’s metal-chelating scaffold given the favorable stacking interaction and the lack of bulky substituents para to DTG’s hydroxyl group. Thus, we chose to model the adenylate for the Q148H/G140S HIV-1 IN-DNA model in a manner most consistent with the pertinent 3S3N density and conformations in 3S3M and 3S3O.

With all relevant crystal structures in hand, MVP and its sequence alignment were used to superimpose the PFV intasome structures from 3L2U, 3OYA, 3S3M, 3S3N and 3S3O onto the HIV-1 IN structure from 2B4J based on their common catalytic cores. To construct the wild-type, Q148H/G140S and N155H HIV-1 IN models, the IN structures from 2B4J, 3OYA, 3S3N and 3S3O were imported into the Insight II program (Insight II Molecular Modeling System, version 2005, Accelrys, Inc, San Diego, CA), and their amino acid sequences were extracted with the Sequences-Extract tool in the program’s Homology module. Next, the NL432 IN sequence of only the catalytic core and hand-edited versions of the latter containing the Q148H/G140S and N155H substitutions were also imported into the modeling program to serve as the amino acid sequences of their respective IN models. The MVP sequence alignment was manually recreated within the Homology module to establish the amino acid correspondences between HIV-1 and PFV IN.

**Wild-Type, Q148H/G140S and N155H HIV-1 IN Models.** The dimeric catalytic core models of wild-type and Q148H/G140S HIV-1 IN were constructed by transferring the atomic coordinates of residues S57 through I208 from both HIV-1 IN monomers in 2B4J to the models, with the exception of residues G140 through E152 of chain A; the atomic coordinates for the latter residues were assigned from the corresponding PFV IN amino acids in 3OYA for the wild-type HIV-1 IN model and 3S3N for the Q148H/G140S HIV-1 IN model. The N155H HIV-1 IN model was built in similar fashion to the first 2 IN models except that the portion of chain A modeled from PFV IN was extended from residues G140 through K159; the atomic coordinates for these amino acids were assigned from the corresponding PFV IN residues in 3S3O. Since a pair of Mg^2+^ ions are not bound at either of the 2 HIV-1 IN catalytic sites in 2B4J, residues D64 and D116 are obviously unlikely to be in metal-bound conformations. Thus, for chain A of all 3 IN models, the rotameric states of D64 and D116 were modified to match those of their PFV IN template, residues D128 and D185, respectively.

Where residue differences occurred between the template portion of PFV IN and that modeled for HIV-1 IN, the rotameric states of the HIV-1 IN amino acids were carefully scrutinized. Two key differences evaluated for the wild-type and N155H HIV-1 IN models involved residues G140 and Q148, which correspond to PFV IN residues S209 and S217, as previously indicated. The PFV IN structures in 3OYA and 3S3O show that S209 and S217 are located at the N- and C-terminal ends of the active-site loop, respectively, and that the side chain of S217 H-bonds to the side chain of S209, which in turn H-bonds to the side chain of H183. This H-bond network appears to help stabilize the loop in a catalytically active state. For the wild-type and N155H IN models, an analogous H-bond network is not possible because of residue G140. However, from a conformational search via Insight’s rotamer library, a conformer for the side chain of Q148 was identified where the residue was able to form an H-bond interaction with the side chain of H114. We speculate that this H-bond helps stabilize the C-terminal end of the active-site loop while residue G140 imparts flexibility at the N-terminal end. As for the Q148H/G140S HIV-1 IN model, the S217H PFV IN structure in 3S3N shows that the side chain of S217H H-bonds to the side chain of S209, which in turn H-bonds to the side chain of H183. Since S217H PFV IN was designed as a surrogate for Q148H/G140S HIV-1 IN, this H-bond network was simply recreated within the mutant IN model by the transfer of atomic coordinates.

At this stage, hydrogen atoms were added to the residues of each HIV-1 IN model using Insight’s Hydrogen command, which also computed the ionization states of the residues at physiologic pH. The IN models were then prepared for refinement by assigning to all atoms the potential atom types and partial charges associated with the CVFF force field via the ForceField command. The entire composite model of each dimeric catalytic core was not energy minimized since it is generally accepted that geometry optimization methods will not bring a model closer to the actual structure and that extensive refinements will actually result in a less accurate model [[12](#_ENREF_12),[13](#_ENREF_13)]. Instead, residues F139 through S153 for the wild-type and Q148H/G140S HIV-1 IN models and residues F139 through K160 for the N155H HIV-1 IN model plus a handful of exceptions for all 3 were structurally refined, but with constraints applied to these calculations.

For all 3 HIV-1 IN models, there was a splice point between residues F139 and G140. Since residue F139 lies at the end of the β5 strand, torsional constraints were imposed on its φ, ψ angles to preserve the continuity of the β-strand, but not hinder the geometry optimization from creating a smooth transition between the β-strand and active-site loop. Distance constraints were imposed between the backbone NH of G140 and the backbone CO of T115 to form an H-bond between the 2 groups as seen crystallographically for the equivalent PFV IN residues. Tethering constraints with a force constant of 50.0 kcal/(mol Å^2^) were also imposed on the heavy atoms of residues I141 through I151 to keep these residues from deviating too far from their starting positions.

For the wild-type and Q148H/G140S HIV-1 IN models, the second splice point was located within the α4 helix between residues E152 and S153. Distance constraints were imposed between the backbone NH and CO groups of E152 and S153 and their H-bonding partners to smooth the helix’s structural fold. A tethering constraint with a force constant of 100.0 kcal/(mol Å^2^) was imposed on the side chain of residue E152 to maintain it in its Mg^2+^-bound rotameric state. For the N155H HIV-1 IN model, the second splice point was located further towards the C-terminal end of the α4 helix between residues K159 and K160. In this case, distance constraints were imposed between the backbone NH and CO groups of K159 and K160 and their H-bonding partners to smooth the helix’s structural fold. A tethering constraint was also imposed on the side chain of residue E152. The N224H mutation in PFV IN not only altered the structure of the α4 helix, but also caused a slight widening of the catalytic site’s base between the α4 helix and β1 strand to accommodate the larger His residue. To model this, residues L63 through C65 were included in the geometry optimization and a template constraint with a force constant of 100.0 kcal/(mol Å^2^) was imposed on residue D64 with the equivalent PFV IN residue D128 from 3S3O serving as the template.

The side chain of residue N117 was also included in the geometry optimization of all 3 HIV-1 IN models, and distance constraints were imposed to form an H-bond with the backbone CO of G140. In the PFV IN structures, the corresponding residue Q186 forms an H-bond with the backbone CO of S209. The side chain of N117 is obviously shorter than that of Q186 by 1 methylene group, but we speculated that the H-bond might form in HIV-1 IN to help stabilize the N-terminal end of the active-site loop in a catalytically active and inhibitor-bound state. Once the geometry optimizations were completed, the H-bonding geometries between the aforementioned groups were non-ideal, suggesting that the H-bond may not actually form and that there may be structural differences between the active-site loops of HIV-1 and PFV IN.

All energy minimization calculations were set up within Insight’s Discover module to act on those portions of the IN models just outlined along with the imposed structural constraints; all other amino acid residues were held fixed. The conjugate gradients algorithm was selected as the minimization method with charges, cross and Morse terms calculated. The convergence criterion for the energy minimization calculations was kept at the default value, and the maximum number of iterations was set to 10,000 steps, which proved sufficient.

**Q148R/K HIV-1 IN Models.** A Q148R HIV-1 IN model was constructed from a copy of the refined wild-type HIV-1 IN model, where residue Q148 was mutated to Arg and its rotameric state modified to match that of the equivalent residue, R322, from the related polynucleotidyl transferase, Tn5 transposase, in 1MUS (sequence alignment not shown). In almost identical fashion to the corresponding Tn5 transposase residues, R322 and E326, the side chain of HIV-1 IN residue Q148R was modeled forming an ionic interaction with the side chain of residue E152. The conformation used for Q148R did create minor steric clashes with the neighboring residue P145. To alleviate this, a geometry optimization of the Q148R HIV-1 IN model was performed using the same options and parameters that were used for the refinement of the other IN models. A distance constraint was imposed between the backbone CO of N144 and the backbone NH of S147 to maintain their H-bond interaction and the structure of the 3_10_ helix. A Q148K HIV-1 IN model was also built in a similar fashion to the Q148R IN model.

**HIV-1 IN-DNA Models in Complex with Mg^2+^ and IN Inhibitors.** The refined dimeric catalytic core models of wild-type, Q148H/G140S and N155H HIV-1 IN were used to assemble a set of 5 metallo nucleoprotein complexes with DTG, RAL or EVG bound at the catalytic site. The first model built was of HIV-1 IN-U5 DNA in complex with 2 Mg^2+^ ions and RAL. Given that the superimposition of the common IN catalytic cores in 2B4J and 3OYA had docked the PFV U5 LTR end in 3OYA onto the wild-type HIV-1 IN catalytic core and that this PFV DNA is a rational template for HIV-1 DNA, a model of the 3′ processed, HIV-1 U5 LTR end was constructed by simply substituting the PFV LTR nucleotides with those of HIV-1 listed in Figure 1B using the Replace Nucleotide command in Insight’s Biopolymer module.

Residue differences between the catalytic sites of HIV-1 and PFV IN were reexamined within the context of the newly assembled HIV-1 IN-DNA model, and the conformations of 3 HIV-1 IN residues were reevaluated based on their potential interactions with viral DNA; these were T66, S153 and K156, which correspond to PFV IN residues I130, R222 and S225, respectively. While PFV IN residue I130 does not form specific interactions with cognate DNA, we reasoned that HIV-1 IN residue T66 likely does given its H-bonding potential. We found from the structure of Tn5 transposase in complex with outside-end DNA in 1MUS that the side chain of T99 forms an H-bond with 1 of the phosphate oxygen atoms of the terminal 3′ guanylate of the transferred DNA strand. Given that T99 is equivalent to HIV-1 IN residue T66, we expect that this HIV-1 IN residue also forms the same type of interaction with cognate DNA affecting the conformation of the terminal 3′ adenylate. Thus, the rotameric state of T66 was modified to match that of T99 allowing for the formation of an H-bond with 1 of the phosphate oxygen atoms of the 3′ adenylate. The intermolecular interactions observed between PFV IN residues R222 and S225 and cognate DNA were not reproducible in the HIV-1 IN-DNA model as the HIV-1 IN residues S153 and K156 differ significantly from their PFV IN counterparts, not to mention the differences in neighboring nucleotides. To establish plausible conformations for S153 and K156, Insight’s rotamer library was used to explore likely conformers for the residues. A rotameric state was selected for S153, where its side chain interacted with nucleotides C3 and T4 (numbering scheme as listed in Figure S1B) of the non-transferred DNA strand, and for K156, where its side chain interacted with nucleotide G3 of the transferred DNA strand. The chosen conformers did create minor steric clashes with their surroundings. To alleviate this, a geometry optimization of the HIV-1 IN-DNA model was performed using the same options and parameters that were used for the refinement of the HIV-1 IN models, but only the side chains of T66, S153 and K156 were refined.

To complete the assembly of the HIV-1 IN-DNA-RAL model, 2 Mg^2+^ ions were added to the catalytic site of chain A of the enzyme coordinated by the acidic residues of the DDE motif. The placement of the metal ions was guided by those present at the active site of PFV IN in 3OYA, but their atomic positions were ultimately set to satisfy the average bond lengths and angles of an octahedrally coordinated Mg^2+^ complex as reported by Bock et al, 1999 [[14](#_ENREF_14)]. Next, RAL was manually docked into the HIV-1 IN catalytic site with its binding mode set nearly identical to that seen in 3OYA. Lastly, the octahedral coordination sphere of the 2 Mg^2+^ ions was completed by adding 3 water molecules to the model guided by the crystallographic waters coordinating the Mg^2+^ ions in 3OYA.

The catalytic core model of wild-type HIV-1 IN was also used to assemble HIV-1 IN-DNA models in complex with EVG and DTG, both of which were constructed in similar fashion to the IN-DNA-RAL model. The IN-DNA-EVG model was built from components taken from the EVG-bound, PFV intasome structure in 3L2U. The PFV DNA served as the template for the HIV-1 U5 LTR end, and EVG and the 2 Mg^2+^ ions bound at the PFV IN catalytic site were used to guide the manual placement of these ligands into the catalytic site of the HIV-1 IN-DNA model. Crystallographic waters were not reported for 3L2U; however, we assumed that the metal cations of the IN-DNA-EVG model are octahedrally coordinated as seen with the more recent PFV intasome structures. As such, the crystallographic waters coordinated to the metal ions at the active site in 3OYA were used to guide the placement of water molecules into the IN-DNA-EVG model. The IN-DNA-DTG model was assembled from components taken from the DTG-bound, PFV intasome structure in 3S3M. Here again, PFV DNA was used as the template for HIV-1 DNA. Dolutegravir and the pair of Mg^2+^ ions bound at the PFV IN catalytic site along with the 3 crystallographic waters coordinated to the metals were used to guide the placement of these ligands into the catalytic site of the HIV-1 IN-DNA model.

A Q148R HIV-1 IN-DNA model was built from the catalytic core model of Q148R HIV-1 IN, but without an inhibitor molecule bound at the catalytic site, although DTG, RAL or EVG could have easily been docked. This was purposely done to highlight the conformation of residue Q148R within the catalytic pocket and its interaction with residue E152. The model for the HIV-1 U5 LTR end used in the assembly of this nucleoprotein complex was built from the PFV DNA in 3S3M. A Q148K IN-DNA model was also built in a similar fashion to the Q148R IN-DNA model.

As for the dimeric catalytic core models of Q148H/G140S and N155H HIV-1 IN, these composite models were used to assemble HIV-1 IN-DNA complexes bound to DTG only. The Q148H/G140S IN-DNA-DTG model was assembled based on the DTG-bound, PFV intasome structure in 3S3N, whereas the N155H IN-DNA-DTG model was assembled based on the DTG-bound, PFV intasome structure in 3S3O. Both of these structural models were constructed in a similar fashion to the inhibitor-bound, HIV-1 IN-DNA models.

**REFERENCE LIST**

1. Adachi A, Gendelman HE, Koenig S, Folks T, Willey R, et al. (1986) Production of acquired immunodeficiency syndrome-associated retrovirus in human and nonhuman cells transfected with an infectious molecular clone. J Virol 59: 284-291.

2. Hare S, Gupta SS, Valkov E, Engelman A, Cherepanov P (2010) Retroviral intasome assembly and inhibition of DNA strand transfer. Nature 464: 232-236.

3. Hightower KE, Wang R, Deanda F, Johns BA, Weaver K, et al. (2011) Dolutegravir (S/GSK1349572) exhibits significantly slower dissociation than raltegravir and elvitegravir from wild-type and integrase inhibitor-resistant HIV-1 integrase-DNA complexes. Antimicrob Agents Chemother 55: 4552-4559.

4. Kobayashi M, Yoshinaga T, Seki T, Wakasa-Morimoto C, Brown KW, et al. (2011) In vitro antiretroviral properties of S/GSK1349572, a next-generation HIV integrase inhibitor. Antimicrob Agents Chemother 55: 813-821.

5. Berman HM, Westbrook J, Feng Z, Gilliland G, Bhat TN, et al. (2000) The Protein Data Bank. Nucleic Acids Res 28: 235-242.

6. Cherepanov P, Ambrosio AL, Rahman S, Ellenberger T, Engelman A (2005) Structural basis for the recognition between HIV-1 integrase and transcriptional coactivator p75. Proc Natl Acad Sci U S A 102: 17308-17313.

7. Hare S, Vos AM, Clayton RF, Thuring JW, Cummings MD, et al. (2010) Molecular mechanisms of retroviral integrase inhibition and the evolution of viral resistance. Proc Natl Acad Sci U S A 107: 20057-20062.

8. Hare S, Smith SJ, Metifiot M, Jaxa-Chamiec A, Pommier Y, et al. (2011) Structural and functional analyses of the second-generation integrase strand transfer inhibitor dolutegravir (S/GSK1349572). Mol Pharmacol 80: 565-572.

9. Steiniger-White M, Rayment I, Reznikoff WS (2004) Structure/function insights into Tn5 transposition. Curr Opinion Struct Biol 14: 50-57.

10. Lambert MH (1997) Docking conformationally flexible molecules into protein binding sites. In Charifson PS, editor. Practical application of computer-aided drug design. New York: Marcel Dekker. pp. 243-303.

11. Afonine PV, Grosse-Kunstleve RW, Echols N, Headd JJ, Moriarty NW, et al. (2012) Towards automated crystallographic structure refinement with phenix.refine. Acta Crystallogr D Biol Crystallogr 68: 352-367.

12. Schonbrun J, Wedemeyer WJ, Baker D (2002) Protein structure prediction in 2002. Curr Opin Struct Biol 12: 348-354.

13. Wieman H, Tondel K, Anderssen E, Drablos F (2004) Homology-based modelling of targets for rational drug design. Mini Rev Med Chem 4: 793-804.

14. Bock CW, Katz AK, Markham GD, Glusker JP (1999) Manganese as a replacement for magnesium and zinc: functional comparison of the divalent ions. J Am Chem Socy 121: 7360-7372.
